# Diet-induced obesity mediated through estrogen-related receptor α is independent of intestinal function

**DOI:** 10.1016/j.jbc.2025.108197

**Published:** 2025-01-16

**Authors:** Kiranmayi Vemuri, Jahangir Iqbal, Sneha Kumar, Alexandra Logerfo, Maria Ibrahim, Eileen White, Michael P. Verzi

**Affiliations:** 1 Department of Genetics, Human Genetics Institute of New Jersey, Rutgers University, Piscataway, New Jersey, USA; 2 Rutgers Cancer Institute of New Jersey, New Brunswick, New Jersey, USA; 3 Ludwig Princeton Branch, Ludwig Institute for Cancer Research, Princeton University, Princeton, New Jersey, USA; 4 Department of Molecular Biology and Biochemistry, Rutgers University, Piscataway, New Jersey, USA; 5 Rutgers Center for Lipid Research, New Jersey Institute for Food, Nutrition & Health, Rutgers University, New Brunswick, New Jersey, USA; 6 NIEHS Center for Environmental Exposures and Disease (CEED), Rutgers EOHSI Piscataway, New Jersey, USA

**Keywords:** estrogen-related receptor α, obesity, intestine, transcription factor, epigenetics, metabolic disease, diet-induced obesity, adipose tissue, intestinal epithelium, obesity therapeutics

## Abstract

Obesity has escalated to epidemic proportions, driving significant advances in therapeutic strategies aimed at combating this condition. The estrogen-related receptor α (ESRRA), a transcription factor, plays pivotal roles in energy metabolism across multiple tissues. Research has consistently shown that the absence of *Esrra* results in notable fat malabsorption and increased resistance to diet-induced obesity. However, existing studies primarily focusing on germline *Esrra* mutants fail to account for tissue-specific roles of ESRRA in obesity. Notably, *Esrra* exhibits high expression in the gastrointestinal tract relative to other tissues. Given the gastrointestinal tract's central role in dietary lipid absorption and metabolism, it is critical to investigate how ESRRA specifically affects this tissue. This study aims to fill this gap by employing advanced mouse genetics and genomics techniques to dissect the impact of ESRRA within the intestine. We also aim to elucidate ESRRA’s specific contributions to diet-induced obesity and refine our understanding of how this transcription factor influences metabolic outcomes in the context of dietary intake.

The growing obesity epidemic has sparked a revolution in the development of therapies targeting obesity. The transcription factor estrogen-related receptor α (ESRRA) has been implicated in obesity as a regulator of mitochondrial metabolism (1, 2) and in the regulation of satiety (3). ESRRA controls the expression of genes involved in mitochondrial biogenesis, gluconeogenesis, oxidative phosphorylation, and fatty acid metabolism across tissues (2, 4, 5, 6). ESRRA is particularly abundant in energy-demanding tissues such as muscle, intestine, brain, adipose, and liver (7, 8). In the liver, *Esrra* knockout leads to reduced blood lipid levels, which is linked to decreased secretion of triglyceride-rich very low density lipoprotein in fasting mice (9). In adipose, ESRRA promotes the differentiation of mesenchymal stem cells into adipocytes. Moreover, ESRR isoforms play a crucial role in brown adipose tissue adaptive thermogenesis (10). In the intestine, whole-body *Esrra* knockout conferred protection against a mouse model of colitis, which was attributed to microbiome alterations, autophagy, and mitochondrial dysfunction (11, 12). Interestingly, despite ESRRA’s importance in energy metabolism, additional studies on *Esrra* mutant mice have shown this receptor is not essential for basic cellular function but is crucial for meeting the energy demands required to respond to physiological and pathological challenges across various tissues (13). However, a significant limitation in most studies involving ESRRA is the use of germline *Esrra* mutants. This broad approach overlooks the tissue-specific, spatial, and temporal nuances of ESRRA's involvement in regulating energy metabolism.

Phenotypes associated with *Esrra* loss in mice include fat malabsorption, reduced fat mass, insulin resistance, and resistance to high-fat diet (HFD)-induced obesity (14, 15). ESRRA’s impact on diet-induced obesity is very interesting in the context of the small intestine, the primary site of dietary lipid metabolism in mammals. Intestinal enterocytes are equipped to metabolize fatty acids through beta-oxidation, a process that not only meets the high energy demands of these cells but also contributes to systemic metabolic flexibility by modulating lipid utilization and storage. The intestine is also a key player in metabolic regulation through its involvement in nutrient absorption, the secretion of gut-derived hormones such as glucagon-like peptide-1 (GLP-1), and microbiota-mediated energy homeostasis (16, 17). These processes highlight the intestine’s crucial role in maintaining systemic energy balance and suggest that intestinal ESRRA might mediate diet-induced obesity. Given the intestine’s central role in metabolism, the gut has become a major player for several therapeutic interventions aimed at treating obesity. Pharmacological agents such as GLP-1 receptor agonists and the lipase inhibitor orlistat, act by modulating intestinal function to influence weight loss (18). Here, we test the hypothesis that intestinal ESRRA plays a critical role in the development of diet-induced obesity. We employ tissue-specific genetic mutants in mice to selectively delete *Esrra* in the gut. Furthermore, we used transcriptomic and epigenomic techniques to map ESRRA-binding sites and identify direct ESRRA target genes in the intestine, providing insights into its role in metabolic regulation.

## Results

### ESRRA is a direct transcriptional activator of genes involved in mitochondrial metabolism in the intestinal epithelium

**Figure 1:**
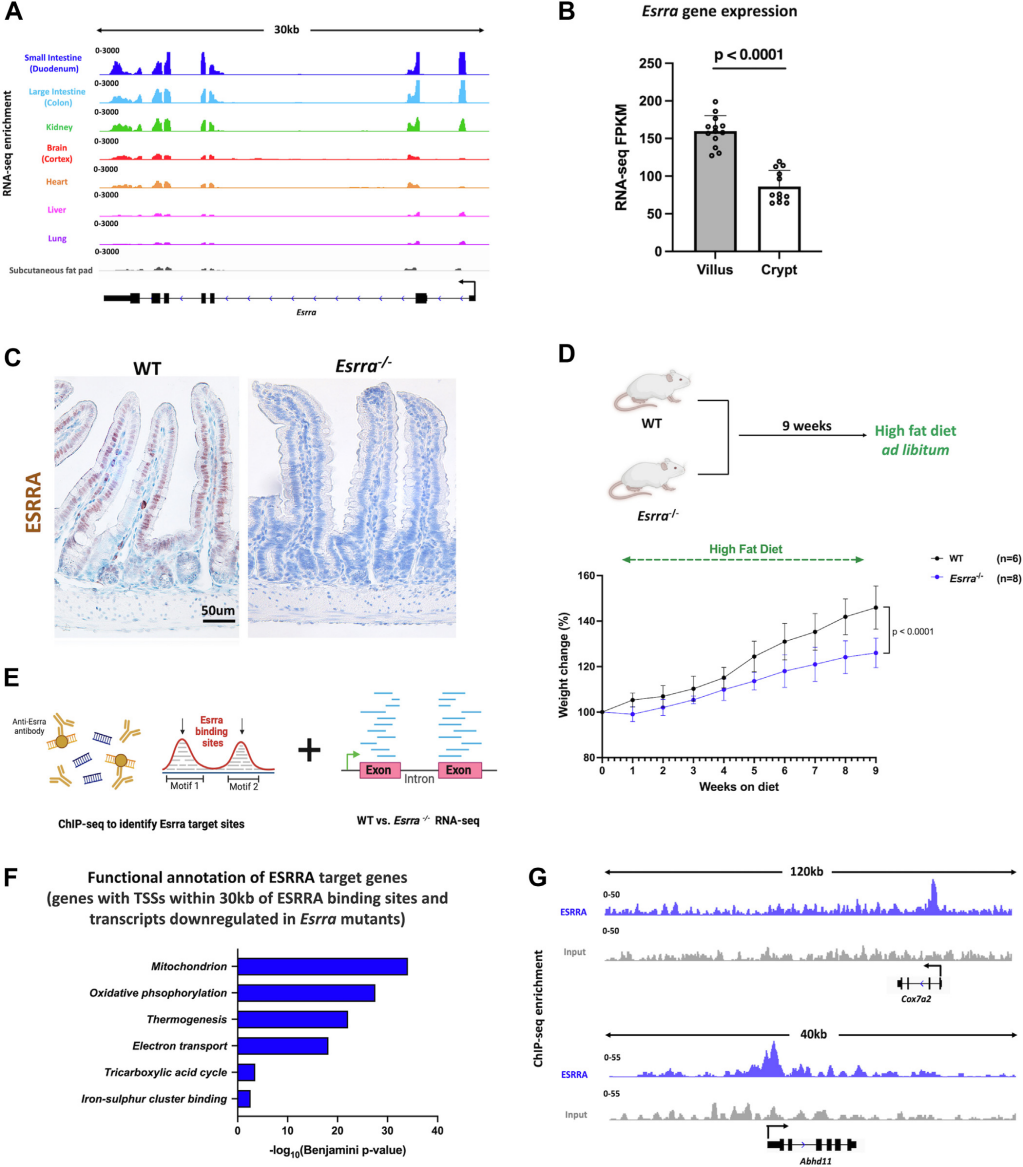
**Characterization of intestinal ESRRA function.***A*, transcript expression of *Esrra* across different tissues (GEO: GSE36025), visualized using IGV. *B*, bar plots show FPKM values for *Esrra* in crypt and villus derived from RNA-seq (GEO: GSE133949). Data is presented as mean ± SD (n = 13 villi and 12 crypts, two-sided Student’s *t* test). *C*, IHC staining of ESRRA protein expression in the proximal small intestine of WT and *Esrra*^*−/−*^ mice. Images are representative of 2 biological replicates per group. *D*, schematic depicting HFD feeding scheme for WT and *Esrra*^−/−^ mice and body weight changes (%) in *Esrra*^*−/−*^ and WT littermates upon 9 weeks of HFD feeding (n = 6–8 biological replicates per group). *E*, schematic showing integration of ChIP-seq and RNA-seq data to identify ESRRA target genes in the intestinal epithelium. *F*, functional annotation (DAVID) of ESRRA target genes (defined as bound and regulated by ESRRA as measured by ChIP-seq; within 30 kb of ESRRA-binding sites; and downregulated in WT *versus Esrra*^−/−^ RNA-seq). *p* values calculated using DAVID. *G*, examples of ESRRA binding to gene loci as illustrated using ESRRA ChIP-seq merged replicate data. ESRRA-bound tracks are in *blue*, input tracks in *gray*, visualized using IGV. ChIP-seq, chromatin immunoprecipitation sequencing; ESRRA, estrogen-related receptor α; FPKM, fragments per kilobase of transcript per million mapped reads; GEO, gene expression omnibus; HFD, high-fat diet; IGV, integrative genomics viewer; IHC, immunohistochemistry.

*Esrra* is highly expressed in the gastrointestinal tract relative to many other tissues (Fig. 1*A*). Hence, we first sought to uncover the biological significance of ESRRA within the small intestinal epithelium. We observed that *Esrra* expression was most pronounced in the differentiated villi compared to the proliferating crypts (Fig. 1, *B* and *C*). We hypothesized that ESRRA would be less important in stem/proliferative functions, and enteroids from intestinal crypts of both WT and *Esrra*^*−/−*^ mice indeed showed no discernible differences in their growth or budding patterns (Fig. S1*A*). To validate a role for ESRRA in promoting diet-induced obesity, we fed both WT and *Esrra*^*−/−*^ mice a HFD. Consistent with previous reports, we observed a clear resistance to weight gain in the *Esrra*^*−/−*^ mice in comparison to their WT littermates, with *Esrra*^*−/−*^ mice weighing 15 to 20% less at 9 weeks of feeding (Fig. 1*D*). Next, we defined ESRRA target genes in the intestinal epithelium by conducting RNA-seq and chromatin immunoprecipitation sequencing (ChIP-seq). We first compared gene expression profiles between WT and *Esrra*^*−/−*^ intestinal epithelia and identified 526 transcripts downregulated in *Esrra*^−/−^ (as determined by DESeq2 (https://bioconductor.org/packages/DESeq2) (19) with a log2 fold change < −0.58 and a *p* value < 0.05; Table S1), which were strongly enriched in annotated mitochondrial functions such as oxidative phosphorylation (Fig. S1, *B*–*D*). This underscores ESRRA's pivotal role as a regulator of mitochondrial activity in the intestine, akin to its functions in other tissues (1, 20, 21). To determine whether ESRRA directly activates these regulated genes, we conducted ChIP-seq and found 1639 genomic regions bound by ESRRA (MACS2 *p* value <10^−5^; Table S2). ESRRA-bound genomic regions were highly enriched in known ESRR DNA-binding motifs, indicating high-quality and reproducible ChIP (Fig. S1, *E* and *F*). Through integration with RNA-seq data, we identified a set of 110 target genes (defined as bound and regulated by ESRRA as measured by ESRRA ChIP-seq; within 30 kb of ESRRA-binding sites; and downregulated in WT *versus Esrra*^*−/−*^ RNA-seq) (Fig. 1*E*). Functional annotation of these direct ESRRA target genes indicated that ESRRA is a major regulator of mitochondrial gene expression in the intestine (Fig. 1*F*). Examples of two illustrative genes that are direct ESRRA targets and have their products localize to mitochondria are *Abhd11* and *Cox7a2* (Fig. 1*G*). In summary, these results suggest that ESRRA primarily functions in the differentiated intestinal epithelium as a direct transcriptional activator of genes involved in mitochondrial metabolism.

### Resistance to HFD-induced obesity is independent of intestinal ESRRA

**Figure 2:**
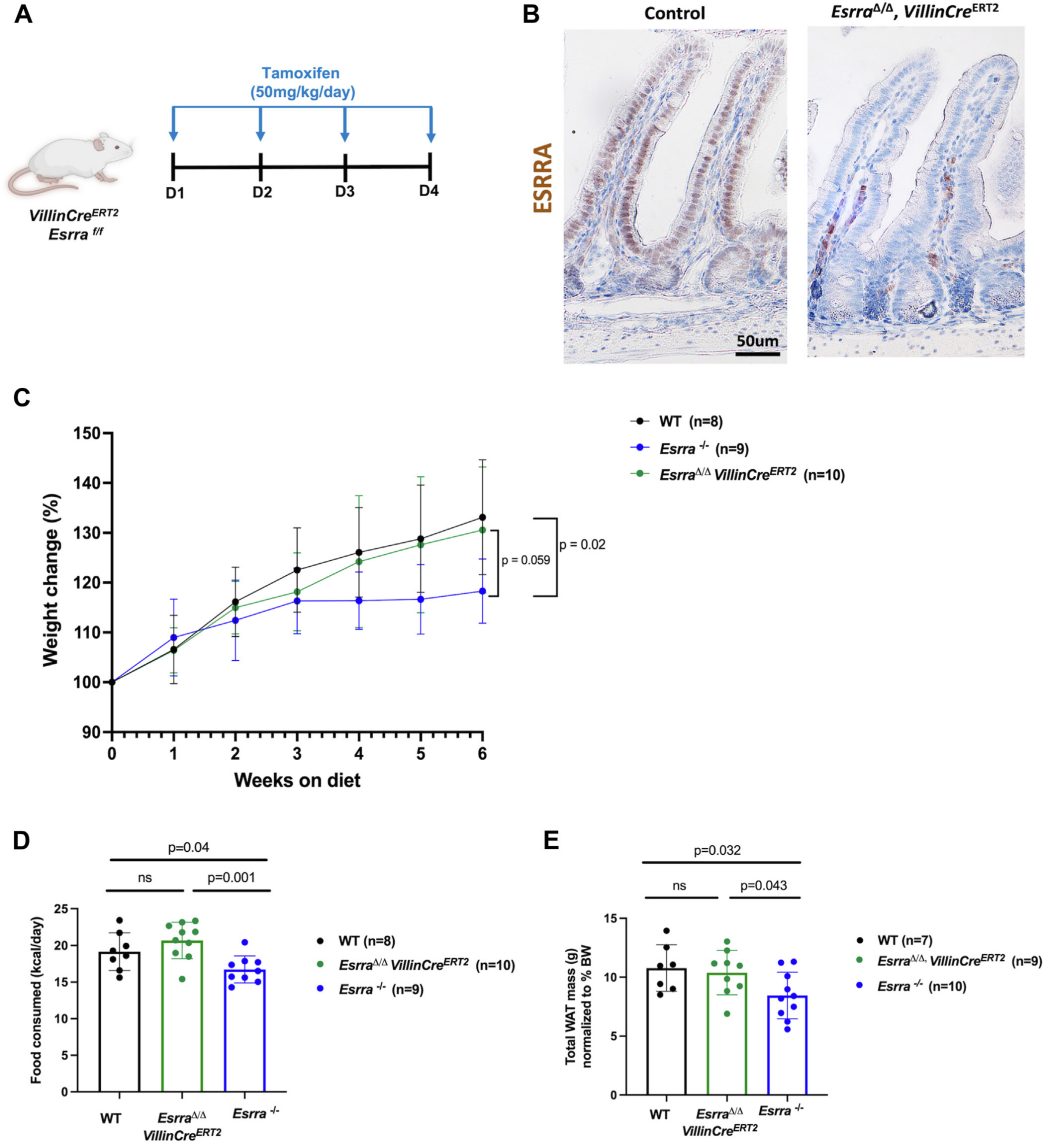
**Effect of intestinal ESRRA on resistance to HFD-induced obesity.***A*, schematic illustrating the tamoxifen injection schedule in *Esrra*^*flox/flox (f/f)*^ mice. *B*, IHC staining of ESRRA protein expression in proximal small intestine of WT and *Esrra*^*Δ/Δ*^ mice. Images are representative of 2 biological replicates per group. *C*, body weight changes (%) in *Esrra*^*−/−,*^*Esrra*^*Δ/Δ*^ and control littermate mice upon 6 weeks of HFD feeding. *Green lines* are indicative of *Esrra*^*Δ/Δ*^ mice, *blue lines* are indicative of *Esrra*^*−/−*^ mice, and *black lines* are indicative of littermate controls (n = 8–10 biological replicates per group). *D*, food intake of HFD in *Esrra*^*−/−*^*, Esrra*^*Δ/Δ*^*,* and control littermate mice (bars represent mean ± SD, n = 8–10 biological replicates per group). *E*, total white adipose tissue (WAT) mass normalized to body weight in *Esrra*^*−/−*^*, Esrra*^*Δ/Δ*^*,* and control littermate mice (bars represent mean ± SD, n = 7–10 biological replicates per group). ESRRA, estrogen-related receptor α; HFD, high-fat diet; IHC, immunohistochemistry.

However, given that loss of *Esrra* occurs across all tissues (Fig. 1) in the germline mutant model, we could not attribute the observed resistance to diet-induced obesity to intestinal ESRRA. Therefore, we created a tamoxifen-inducible *Esrra* deletion model, integrating *VillinCre*^*ERT2*^ and *Esrra*^*flox/flox*^^*(f/f)*^, to target *Esrra* loss to the intestinal epithelium (Fig. 2*A*). Intestine-specific loss of *Esrra* (referred to as *Esrra*^*Δ/Δ*^) was confirmed by immunohistochemistry (Fig. 2*B*), western blotting (Fig. S2*A*), and mRNA expression analysis (Fig. S2*B*). We also quantified ESRRA protein levels in the small intestine, liver, and white adipose tissue under both chow and HFD conditions. Interestingly, we observed an increase in ESRRA protein levels in the intestine of HFD-fed mice (Fig. S2, *C*–*E*). These findings suggested a potential intestine-specific role for ESRRA in response to HFD. We therefore challenged the *Esrra*^*Δ/Δ*^ mice with HFD for 6 weeks alongside littermate *Esrra*^*−/−*^ and vehicle-treated controls. This duration was selected to capture the early onset of diet-induced obesity while avoiding potential confounding effects of prolonged HFD feeding and larger differences in adiposity. Interestingly, while we again observed resistance to HFD-induced obesity in *Esrra*^*−/−*^ mice, the intestine-specific mutants did not exhibit resistance to obesity, suggesting ESRRA in the intestine does not contribute to diet-induced weight gain (Fig. 2*C*). We also tested whether the mutants exhibited differential energy expenditure or feeding behaviors upon HFD consumption. We saw no significant differences in energy expenditure or related parameters between *Esrra*^*−/−*^ and WT mice (Fig. S2*F*). This is consistent with previous studies showing no changes in energy expenditure following germline *Esrra* loss, although this was observed under chow feeding conditions (15). However, we did observe that *Esrra*^*−/−*^ mice consumed less food, while *Esrra*^*Δ/Δ*^ mice displayed food intake comparable to the controls (Fig. 2*D*). Consistent with the body weight and feeding behaviors, we noted less white adipose tissue in the germline mutant mice (Fig. 2*E*). Taken together, our findings suggest that intestinal ESRRA does not govern resistance to HFD-induced obesity and supports investigation in other tissues to define the functions of ESRRA in diet-induced obesity.

## Discussion

Obesity is a multifactorial condition, influenced by a combination of genetic, environmental, behavioral, and physiological factors. Understanding the transcriptional regulation of dietary lipid metabolism opens avenues for therapeutic interventions. Modulating transcription factors in specific tissues offers a unique opportunity to intervene at the gene expression level, minimizing off-target effects and addressing the root molecular causes of diet-induced obesity. Our results suggest that the transcription factor ESRRA might contribute to diet-induced obesity *via* its role in tissues other than the small intestine, such as adipose, muscle, and/or brain. The pan-tissue expression of *Esrra* underscores the importance of precise targeting in therapeutic interventions. Our metabolic study found no significant differences in energy expenditure between *Esrra*^*−/−*^ and WT mice fed a HFD for 6 weeks. Similar results were observed in *Esrra*^*−/−*^ and WT mice-fed chow diet (15). These findings suggest that ESRRA may not play a major role in regulating systemic energy expenditure in response to dietary changes, though further studies are needed to better understand the specific contributions of ESRRA to energy metabolism and mitochondrial function in other tissues.

Although mutations in *Esrra* trigger resistance to obesity, recent work has increasingly focused on developing agonists of ESRRA. Recent reports have shown that diabetics and individuals with a family history of diabetes and metabolic conditions have reduced oxidative phosphorylation capacity in their muscles (22, 23). These findings support the hypothesis that drugs boosting aerobic respiration in muscle might improve diabetes. It is well-established that aerobic exercise, one of the best nonpharmacologic interventions for ameliorating diabetes, increases mitochondrial content and promotes oxidative phosphorylation gene expression. This highlights the potential of using ESRRA, a major mitochondrial regulator, to control the oxidative phosphorylation transcriptional programs which are altered in diabetic muscle (24, 25). Consequently, efforts have increasingly focused on identifying agonists of ESRRA. Studies have identified forskolin, phorbol 12-myristate 13-acetate, and several statins as agonists for ESRRA or ESRRA/PPARGC1A dimers (26, 27). Additionally, a synthetic ESRRA agonist, SLU-PP-332, has been shown to induce an ESRRA-specific acute aerobic exercise genetic program by increasing oxidative skeletal muscle fibers and improving exercise endurance in mice (28). However, while activation of ESRRA in skeletal muscle can ameliorate diabetes and improve exercise endurance, it might have undesirable effects in other tissues, as ESRRA is a biomarker for poor prognosis in multiple cancers (29, 30, 31, 32, 33, 34). Therefore, it has become increasingly important for future research to delineate the tissue-specific roles of ESRRA and to determine how it can be targeted pharmacologically without causing off-target effects. Our work carefully delineates the intestinal role of ESRRA in metabolic regulation and suggests future studies focus on ESRRA in other tissues for roles in systemic metabolic processes such as energy balance and food intake regulation. Studies have shown that shRNA-mediated silencing of *Esrra* in the medial prefrontal cortex of the brain leads to reduced feeding and resistance to HFD-induced obesity, suggesting that ESRRA promotes weight gain through behavioral control in the central nervous system (3). This is notable because the brain has been identified as a major target for GLP-1 receptor agonists. While GLP-1 from the gut is rapidly degraded and is believed to have minimal impact on appetite, both the hormone and its receptors are naturally found in various brain regions (35). Studies suggest that GLP-1 drugs cross the blood-brain barrier to control appetite (36, 37). This raises important questions about the role of the gut–brain axis in appetite regulation and metabolic control, and whether ESRRA may interact with GLP-1 signaling to mediate these processes. Knowledge about tissue-specific ESRRA function in the intestine provides valuable directions for future efforts to combat the growing global burden of metabolic diseases. While the goal of our current study was to narrow the contributions of ESRRA to the onset of obesity, future studies will be needed to assess its role in more advanced stages of obesity.

## Experimental procedures

### Animals

*Esrra*^*f/f*^ (JAX stock #034713) (38) and *Esrra*^−/−^ (MGI:3604552, Jackson Labs) mice were bred to integrate the *Villin-Cre*^*ERT2*^ transgene (39). Mice with *Villin-Cre*^*ERT2*^ and heterozygous for *Esrra*^*f/−*^ alleles were bred to generate *Esrra*-floxed (*Esrra*^*f/f*^) and *Esrra*-null (*Esrra*^−/−^) littermate mice. Experimental *Villin-Cre*^*ERT2*^ *Esrra*^*f/f*^ mutant mice (6 weeks old) received 4 intraperitoneal injections of tamoxifen at 50 mg/kg/day (Sigma, T5648). Cage mate controls were given corn oil vehicle (Sigma, C8267). All groups were fed HFD (Research Diets, D12492) *ad libitum*, starting 4 days after the final tamoxifen or oil injection. Mice were weighed weekly in a blinded manner. Following HFD, mice were single-housed for 7 days in wire-bottom metabolic cages to monitor food consumption. Food intake was measured every 48 h.

For adipose tissue weight measurements, mice were returned to regular cages for 1 week after the metabolic cage period before being sacrificed to collect the duodenum and adipose tissue samples. For RNA-seq experiments, duodenum was collected from *Esrra*^*−/−*^ and littermate control mice fed a HFD for approximately 8 weeks. For ChIP-seq experiments, duodenal villi were collected from C57BL/6 mice fed regular chow. Both male and female mice were used in all *Esrra*^−/−^-related experiments, whereas for experiments involving *Esrra*^*f/f*^ mutant mice, only males were used. All protocols and experiments were approved by the Rutgers Institutional Animal Care and Use Committee.

### Indirect calorimetry in mice

For indirect calorimetry, WT and *Esrra*^*−/−*^ mice were fed a HFD for 3 weeks and single-housed for 48 to 72 h prior to the start of the experiment. During the experiment, mice were single housed in the Promethion Metabolic Cages system (Sable Systems International) under a 12 h light-dark cycle for 4 days. The first 24 h of data collection was removed from analysis for acclimation. Energy expenditure, oxygen consumption, carbon dioxide production, and respiratory exchange ratio were measured. Raw data files were collected and processed by MacroInterpreter 3, which produced standardized output formats for the metabolic variables of interest at each cage. The processed data was analyzed by CalR: A Web-based Analysis Tool for Indirect Calorimetry Experiments (https://calrapp.org) as described previously (40).

### Immunohistochemistry

Intestinal tissues were fixed overnight in 4% paraformaldehyde at 4 °C, dehydrated through a series of ascending alcohols and xylene, and paraffin-embedded. Paraffin sections (5 μM thickness) were processed through heat-mediated antigen retrieval in citric acid buffer (pH 6.0) for 30 mins, followed by staining with primary antibody (ESRRA, Santa Cruz sc-65718, 1:1000 dilution). A biotinylated secondary antibody was applied and the Vectastain ABC horseradish peroxidase Kit (Vector Labs, PK-6101) was used for detection. The slides were counterstained with hematoxylin, mounted, and examined using a Lumenera INFINITY3 camera and Infinity Analyze imaging software (v6.5.6; www.teledynevisionsolutions.com/products/infinity-analyze).

### Epithelial cell isolation

The duodenum was dissected and opened longitudinally to wash the lumen and expose epithelial cells. Tissue was cut into 1-inch pieces and rinsed with ice-cold PBS. Tissue was treated with 3 mM EDTA/PBS for 35 min with the EDTA solution refreshed at the 5- and 10-min marks. Mechanical force was applied to detach the epithelial cell layer from the underlying mesenchyme. Epithelial cells were either collected in entirety or crypts and villi were collected separately using a 70 μM cell strainer. Cells were washed twice with ice-cold PBS and pelleted by centrifugation at 300 rcf at 4 °C.

### Cell lysis and protein extraction

Primary intestinal epithelial cells were processed in radioimmunoprecipitation assay (RIPA) buffer (150 mM sodium chloride, 50 mM Tris pH 8.0, 1% NP-40, 0.5% sodium deoxycholate, and 0.1% SDS in water) to extract total protein. RIPA buffer was spiked with inhibitors to achieve final concentrations of: 1× protease inhibitor, 1 mM PMSF, 1 mM sodium orthovanadate, 10 mM sodium fluoride, and 10 mM sodium butyrate. Samples were sonicated for 2 to 3 min at 4 °C with 30 s on-off pulses at 30% amplitude using a Q800R3 sonicator (QSonica Sonicators). The sonicate was centrifuged at 15,000 rpm for 10 min at 4 °C, and the resulting supernatant containing protein was used for immunoblotting assays. Protein was quantified *via* the Pierce BCA Protein Assay Kit (Thermo Fisher Scientific, 23225), using a SpectraMax M2 plate reader (Molecular Devices) and SoftMax Pro software (Molecular Devices; https://www.moleculardevices.com).

### Immunoblotting

Protein lysates were mixed with loading dye and 0.1 M DTT and heated at 95 °C for 10 min 20 μg of protein for each sample was loaded into a NuPAGE 4 to 12% Bis-Tris polyacrylamide gel (Thermo Fisher Scientific, NP0336BOX) and run in NuPAGE MES SDS running buffer (Thermo Fisher Scientific, NP0002). Proteins were wet-transferred onto polyvinylidene fluoride membrane, which was subsequently blocked in 5% nonfat milk for 1 h at room temperature and incubated overnight with primary antibody in blocking buffer (ESRRA, Santa Cruz sc-65718, 1:1000 dilution). After washing in tris-buffered saline with 0.1% Tween 20 detergent, membranes were incubated with a secondary antibody conjugated to horseradish peroxidase for 1 h at room temperature. Chemiluminescence substrate (Ultra Digital-ECL substrate solution, KindleBio, R1002) was used to detect and image protein (KwikQuant Imager, Kindle Biosciences, D1001).

### Intestinal organoid culture

Primary crypt-derived organoids from *Esrra*^*−/−*^ mice and their WT littermate controls were isolated from the duodenum and cultured in Cultrex reduced growth factor basement membrane extract R1 (BME-R1) (R&D Systems, 3433-005-R1) according to established methods (41). Images were captured using a Zeiss Axiovert 200 Microscope (Zeiss).

### RNA extraction and cDNA synthesis

Cells were isolated from mouse duodenum as described and processed for RNA extraction using Trizol (Invitrogen, 15596018). RNeasy Micro Kit (Qiagen, 74004) was used to extract RNA according to the manufacturer’s instructions. For complementary DNA (cDNA) synthesis, 1 μg of RNA was processed using the SuperScript III First-Strand Synthesis SuperMix, as per manufacturer’s instructions (Thermo Fisher Scientific, 18080-400).

### Reverse transcription quantitative PCR

Reverse transcription quantitative PCR using Thermo Fisher Scientific’s QuantStudio Real-Time PCR System in a 384-well plate loaded with SYBR green (Thermo Fisher Scientific, 4309155), cDNA, and reaction mix containing 0.5 μM final primer concentrations. Primer sequences used are listed in Table S3. The amplification conditions used were as follows: 50 °C for 2 min, 95 °C for 10 min, followed by 40 cycles of 95  °C for 15 s, and 60  °C for 1 min. Hypoxanthine-guanine phosphoribosyl transferase 1 (*Hprt1*) was used as an internal control. The 2^−ΔΔCt^ method was applied to calculate the fold change of the relative transcript level.

### RNA-seq data analysis

RNA samples from WT and *Esrra*^*−/−*^ duodenal epithelia were sent to BGI for transcriptome library preparation and paired-end RNA-seq (20 million reads). Raw sequencing reads (fastq) were quality checked using fastQC (v.0.11.3) (42) and aligned to mouse mm9 reference genome using Kallisto (v2.1.0) (43). DESeq2 (v.2.2.1; https://bioconductor.org/packages/DESeq2) (19) was used to calculate the fragments per kilobase of transcript per million mapped reads and for differential expression analysis. Genes with fragments per kilobase of transcript per million mapped reads  > 1 were used for further analysis. Gene set enrichment analysis (GSEA v.3.0) (44) was performed on the preranked gene list. Gene ontology analysis was performed with DAVID (v.6.8) (45, 46). RNA-seq data from gene expression omnibus datasets GSE36025 (47) and GSE133949 (48) were reanalyzed to examine the expression levels of *Esrra*.

### ChIP-seq and data analysis

ChIP-seq was conducted as previously described with slight modifications (49). Briefly, villus cell pellets were cross-linked in 2 mM disuccinimidyl glutarate (Thermo Fisher Scientific, 20593) for 45 min, and then further cross-linked in 1% formaldehyde (Sigma, F8775) for 20 min at room temperature, pelleted, and washed with ice-cold PBS. Fixed cells were lysed in 3× volume of lysis buffer (1% SDS, 10 mM EDTA, 50 mM Tris–HCl pH 8.0, 1× protease inhibitor cocktail (G-Biosciences, 786-433) and sonicated for 8 min (30 s on-off, 20% amplitude) using a QSonica Q800R3 sonicator to shear chromatin to 300-600 bp. Protein A/G beads (Invitrogen, 10001D and 10004D) were washed with 1% bovine serum albumin in PBS and preloaded with 5 μg of anti-ESRRA antibody (Santa Cruz, sc-65718). Antibody-conjugated beads were incubated with sheared chromatin in dilution buffer with SDS at a final concentration of 0.24% on a rotator at 4 °C overnight. Antibody-conjugated beads were washed five times with RIPA buffer (50 mM Hepes pH 7.6, 1 mM EDTA, 0.7% sodium deoxycholate, 1% NP-40, and 0.5 M LiCl), and once with TE buffer (10 mM Tris, 0.1 mM EDTA). Cross-links were reversed in 0.1 M NaHCO3 and 1% SDS overnight at 65 °C. DNA was column purified using the MinElute PCR purification kit (Qiagen, 28004) and quantified using Picogreen (Invitrogen, P7581). Libraries were prepared using the ThruPLEX DNA-Seq kit (Takara, R400675) and DNA Unique Dual Index Kit (Takara, R400666). Paired-end sequencing of ChIP-seq libraries was performed to a depth of 30 to 50 million reads.

FastQC (v0.11.3; https://www.bioinformatics.babraham.ac.uk) (42), alignment to the mm9 reference genome using bowtie2 (v2.2.6; https://bowtie-bio.sourceforge.net/bowtie2/index.shtml) (50), and subsequent processing were done as previously described (49). Replicates were merged, sorted, and indexed using Samtools (v0.1.19) (51). DeepTools2 bamCoverage (v2.4.2) (52) was used to generate reads per kilobase of transcript per million reads mapped-normalized bigwig files. The Integrative Genomic Viewer (v2.8.13) (53) was used to visualize normalized bigwig tracks. Enriched ontologies were identified with DAVID (v2021) (45, 46) or GREAT (v4.0.4) (54). For generation of heat maps, deepTools2 (v2.4.2) (52) computeMatrix, and plotHeatmap were used.

## Data availability

ChIP-seq and RNA-seq data have been deposited in gene expression omnibus at accession numbers GSE269824 and GSE269825, respectively.

### Statistical analysis

Data is presented as mean ± SD and graphed using GraphPad Prism (v9.50; https://www.graphpad.com), with individual replicates plotted. Statistical analyses were conducted with *t* tests or two-way ANOVA with *post hoc* analyses, unless otherwise specified.

## Supporting information

This article contains supporting information.

## Conflict of interest

The authors declare that they have no conflicts of interest with the contents of this article.
