# Supplementary material for: Diet-induced obesity mediated through estrogen-related receptor α is independent of intestinal function
Source: J Biol Chem. 2025 Jan 16;301(2):108197. doi: 10.1016/j.jbc.2025.108197 (PMC11849689; doi:10.1016/j.jbc.2025.108197)
Supplement: Supporting Information [file mmc4.pdf]

Diet-induced obesity mediated through estrogen-related receptor α is independent of intestinal function

Kiranmayi Vemuri,[1] Jahangir Iqbal,[1] Sneha Kumar,[1] Alexandra Logerfo,[1] Maria Ibrahim,[2,4] Eileen White,[2,3,4] and Michael P. Verzi,[1,2,5,6] *

[1] Department of Genetics, Human Genetics Institute of New Jersey, Rutgers University, Piscataway, NJ 08854, USA
[2] Rutgers Cancer Institute of New Jersey, New Brunswick, NJ 08903, USA
[3] Department of Molecular Biology and Biochemistry, Rutgers University, Piscataway, NJ 08854, USA.
[4] Ludwig Princeton Branch, Ludwig Institute for Cancer Research, Princeton University, Princeton, NJ 08544, USA.
[5] Rutgers Center for Lipid Research, New Jersey Institute for Food, Nutrition & Health, Rutgers University, New Brunswick, NJ 08901, USA
[6] NIEHS Center for Environmental Exposures and Disease (CEED), Rutgers EOHSI Piscataway, NJ 08854, USA
*Correspondence: verzi@biology.rutgers.edu

List of Supplementary Figures

Fig. S1. Further characterization of intestinal ESRRA function using WT and *Esrra* $^{-/-}$ mice.
Fig. S2. Functional insights into the metabolic role of ESRRA

List of Supplementary Tables

Table S1. Differential expression analysis results for WT vs *Esrra* $^{-/-}$ intestinal epithelia, as determined by RNA-seq
Table S2. Coordinates for ESRRA binding sites in intestinal villi, as determined by ESRRA ChIP-seq.
Table S3. List of primer sequences used in this study.

Supplementary Figures

Fig. S1

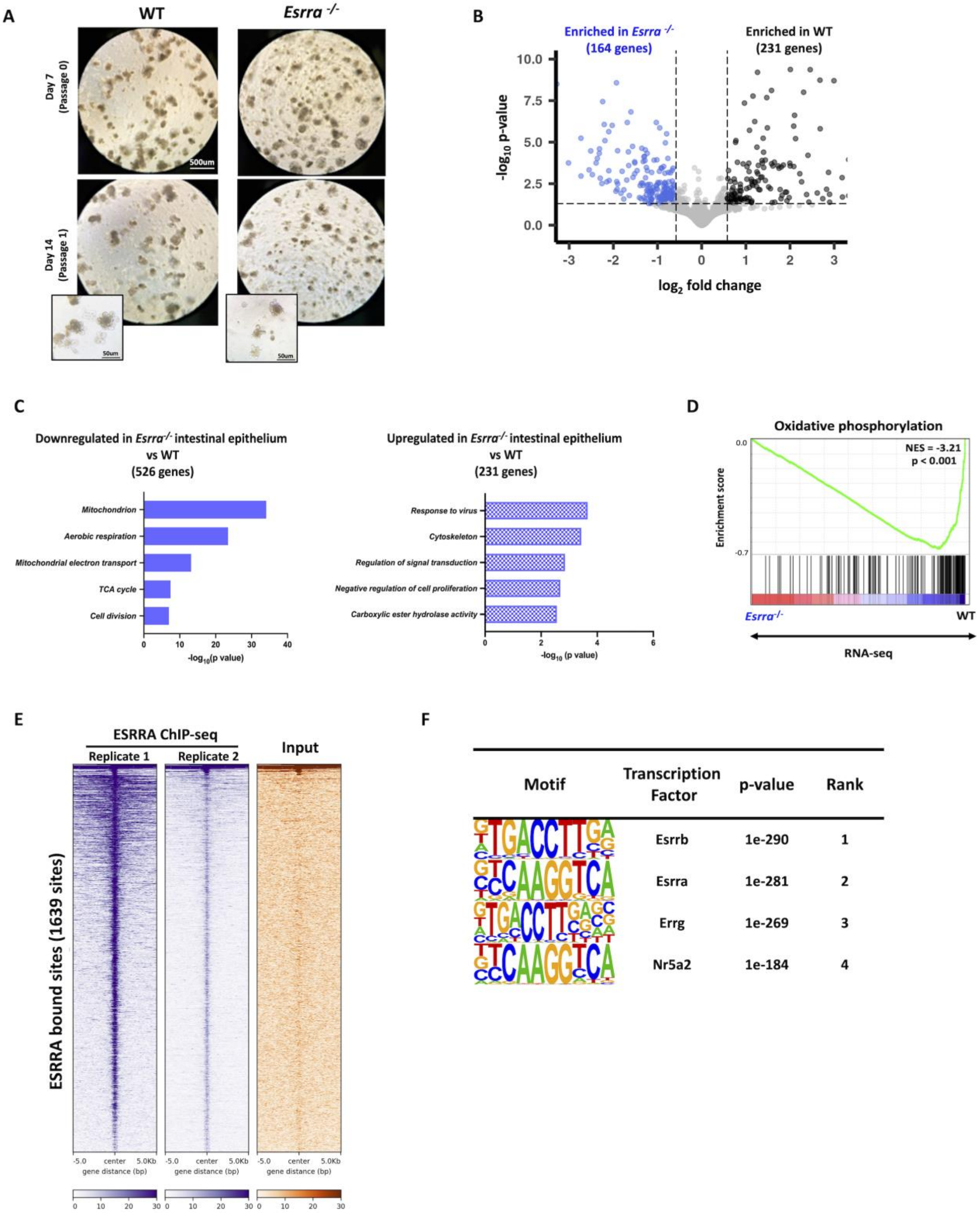

Fig. S1. Further characterization of intestinal ESRRA function using WT and *Esrra*$^{-/-}$ mice. **(A)** Primary organoid cultures isolated from WT and *Esrra* $^{-/-}$ mice. Images are representative of 3 biological replicates. Scale bars are at 500 μm and 50 μm for inset images. **(B)** Volcano plot of differential gene expression between WT and *Esrra*$^{-/-}$ intestinal epithelial cells (n = 3 biological replicates per group). Differential expression was called with DESeq2 (l2FC > 0.58 or < - 0.58, p-value < 0.05). Genes enriched in *Esrra* $^{-/-}$ intestinal epithelia are depicted as blue points and genes enriched in WT-enriched epithelia are depicted as black points. **(C)** Functional annotation (DAVID) of genes enriched in WT- and *Esrra*$^{-/-}$ intestinal epithelia. p-values were calculated using DAVID. **(D)** Gene set enrichment analysis (GSEA) shows reduction of genes with mitochondrial functions upon *Esrra* loss in the intestinal epithelium (Kolmogorov-Smirnov test, p < 0.001). **(E)** Heatmap shows the distribution of ESRRA signal across gene loci for 1639 ESRRA bound sites (MACS2 p-value < 10^{-5}). **(F)** ESRRA DNA-binding motifs identified using HOMER. p-values were calculated with HOMER.

Fig. S2

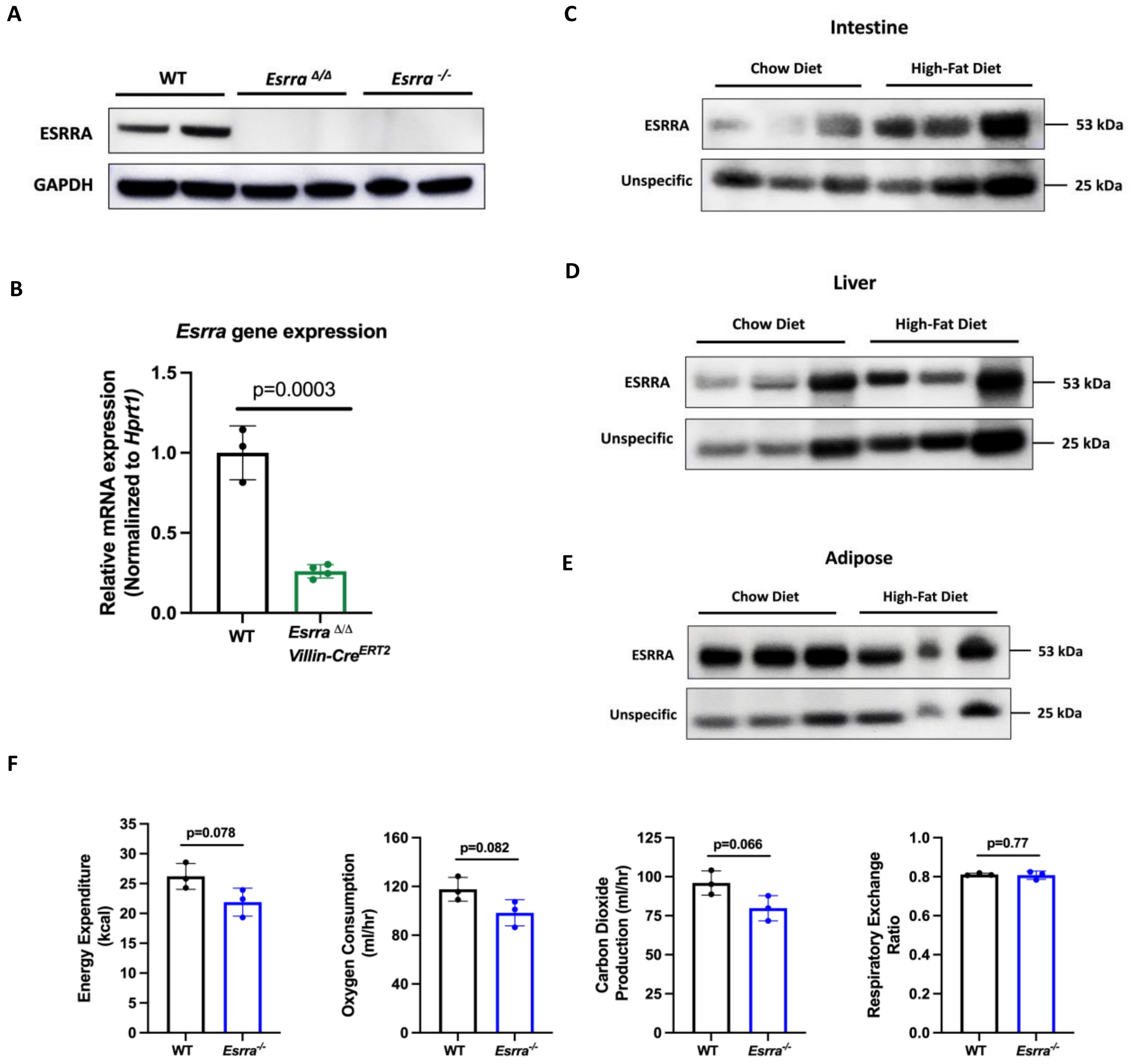

Fig. S2. Functional insights into the metabolic role of ESRRA. (A) Immunoblots showing ESRRA and loading control GAPDH expression in WT, *Esrra*$^{\Delta/\Delta}$ intestinal mutants and *Esrra* $^{-/-}$ germline mutants (n= 2 biological replicates per group). **(B)** Gene expression analysis of *Esrra* in duodenum of WT and *Esrra*$^{\Delta/\Delta}$ intestinal mutant mice (n = 3-4 biological replicates per group, normalized to the housekeeping gene, *Hprt1*; unpaired *t*-test, bars represent mean ± SD). **(C)** Immunoblot shows intestinal ESRRA expression in mice fed chow or high-fat diets in WT mice (n = 3 biological replicates per diet). **(D)** Immunoblot shows liver ESRRA expression in mice fed chow or high-fat diets in WT mice (n = 3 biological replicates per diet). **(E)** Immunoblot shows ESRRA expression in white adipose tissue of mice fed chow or high-fat diets in WT mice (n = 3 biological replicates per diet). **(F)** Indirect calorimetry measurements showing energy

expenditure rates, oxygen consumption, carbon dioxide production and the respiratory exchange ratios in WT and *Esrra* $^{-/-}$ mice (n = 3 biological replicates per group).
